# Deviations in Circulating TNF**α** Levels and TNF**α** Production by Mononuclear Cells in Healthy Human Populations

**DOI:** 10.1155/2011/972609

**Published:** 2011-08-10

**Authors:** T. Mózes, I. Baráth, K. Gornicsar, A. Grosz, Ta. Mózes, Cs. Göndöcs, P. Széphalmi, K. Gaál, E. Madarász

**Affiliations:** ^1^Health Centre, Ministry of Defense, Róbert Károly krt44, 1134 Budapest, Hungary; ^2^Department of Trauma, Péterfy Hospital, Péterfy Sándor utca 14, 1076 Budapest, Hungary; ^3^Department of Urology, Péterfy Hospital, Péterfy Sándor utca 14, 1076 Budapest, Hungary; ^4^Department of Psychology, Eötvös Lorand University, Izabella utca 46, 1064 Budapest, Hungary; ^5^Department of Rehabilitation, Péterfy Hospital, Péterfy Sándor utca 14, 1076 Budapest, Hungary; ^6^Department of Trauma, Dorottya Kanizsai Hospital, Szekeres József utca 2-8, 8800 Nagykanizsa, Hungary; ^7^Institute of Experimental Medicine of Hungarian Academy of Sciences, Szigony utca 49, 1083 Budapest, Hungary

## Abstract

*Objectives*. Tumor necrosis factor alpha (TNF*α*) plays a pivotal role in the inflammatory host response. The serum-level of TNF*α* and the production of TNF*α* by lympho/monocytes, however, seem to show high individual variations. The goal of the present study was to investigate the variations and inducibility of TNF*α*-activity in two age-groups of healthy volunteers. *Methods*. Sixty elderly, healthy volunteers were studied. These persons were free of malignant diseases, and within three months, they did not have any trauma or inflammatory disease and were not taking any steroids or nonsteroid anti-inflammatory drugs. Thirty young volunteers were also included. Blood samples were taken; lympho/monocytes were separated and cultured with or without endotoxin (LPS) stimulation. Serum and culture supernatant TNF*α* levels were determined by bioassay using WEHI 164 cells. *Results*. The results indicated significant individual variations in TNF*α* levels of healthy volunteers irrespective of age. Subgroups with low, middle, and high serum TNF-levels were distinguished. In about 50% of volunteers with low serum-TNF*α* activity, LPS stimulation failed to increase the TNF*α* production by isolated lympho/monocytes. *Conclusion*. Our data suggest a chance to select individuals with enhanced sensitivity for septic complications.

## 1. Introduction

Endotoxin (lipopolysaccharide (LPS) of the outer membrane of Gram-negative bacteria) potently stimulates human monocytes to release several substances with important biological activities, including interleukin 1 (IL-1), tumor necrosis factor alpha (TNF*α*), and prostaglandin E_2_ (PGE_2_) [1]. These factors induce a multitude of biological responses of importance in homeostasis, in host defensive mechanisms, and probably in the pathogenesis of several diseases [1, 2]. 

It has been reported that monocytes can be handled in vitro without “spontaneous” activation of monokine secretion, but extremely low concentrations of LPS can induce significant secretion of IL-1 TNF*α* and PGE_2_ from human monocytes [3]. It was demonstrated that the secretion of IL-1-TNF*α* and TNF*α*-PGE_2_ were strongly correlated and that interindividual differences in monokine and PGE2 secretions do occur [3]. 

Cyclo-oxygenase (CO) metabolites such as PGE_2 _ have been shown to inhibit TNF*α* production at high concentrations, presumably by augmenting cAMP levels in the cells. Low concentrations of PGE_2_ however, appear to stimulate guanylate cyclase and result in augmented TNF*α* production. TNF*α* mRNA accumulation is also inhibited by PGE_2_, an effect associated with decreased TNF*α* transcription [4]. 

The circulating level, activity, and the rate of production of TNF*α* are strongly regulated by both inherent and environmental factors. Genetic variations in the promoter region of *tnfa* [5], the activity of TNF-processing metalloproteinases [6], the level of other cytokines [3, 4] and soluble TNF receptors [7], as well as the density and the signaling cascade of cell-bound TNF receptors [8] are all involved in the establishment of the actual TNF*α* activity. The multiplicity of regulatory pathways leads to fluctuations and large interindividual differences. Even if the reasons behind the individual variations are not completely explored, TNF*α* status might be estimated by immunochemical determination of the circulating protein level or by evaluating the rate of transcription from *tnfa *genes. The physiologically active cytokine level, however, can be best approached by bioassays, those indicating the biological effectiveness of the substance. Each approach has advantages and disadvantages. In the presented study, bioassays on WEHI-164 cells were used to evaluate the biologically active level of TNF*α*.

The aim of the present study was to investigate the possible interindividual differences in serum TNF*α* activities and in TNF*α* production by peripheral blood mononuclear cells (PBMCs) in response to LPS and to relate such differences to the ages of healthy individuals.

## 2. Materials and Methods

Sixty elderly volunteers were included in this experiment. Subjects were residents in an 80-bed rehabilitation facility in a general hospital. Exclusion criteria were malignant disease, inflammation, infection, trauma within three months, and taking nonsteroid anti-inflammatory drugs and/or antibiotics. Thirty young volunteers (both genders) were collected from hospital staff with the same exclusion criteria. Since IL-1 serum levels vary in relation to ovulation [9], young females were studied on day 1 and 14 of menstrual cycle. Seven females of 30 volunteers were included. After obtaining informed consent, blood samples were taken from donors who had no physical exercise on the morning of blood sampling and had normal temperature. The subjects studied are described in Table 1. 

### 2.1. Specimen Collection

A Vacutainer system was used for taking blood. Venous blood was collected in EDTA tubes. For best results, blood was processed for PBMCs within 2 hours. A separate tube was used for obtaining serum samples, which were stored at −20°C until TNF*α* assay. 

### 2.2. Isolation of Peripheral Blood Mononuclear Cells (PBMCs)

Seven mL whole blood was carefully layered onto the 7.0 mL of HISTOPAQUE-1077 (Sigma, Hungary) in a 50-mL conical tubes and were centrifuged at 400 g for 10 minutes at room temperature. After centrifugation, the opaque interface was carefully transferred into a clean conical centrifuge tube and mixed with 10 mL isotonic phosphate-buffered saline (PBS; pH = 7.2). After centrifugation (250 g for 10 minutes), the cell pellet was resuspended in 12 mL PBS. The procedure was repeated 3-times in order to remove HISTOPAQUE contamination, then the cells were resuspended in RPMI 1640 with Hepes (Gibco, Paisley, UK) and L-glutamine, 0.8 × 10^−3^ mol/L supplemented with 5% bovine serum (referred to as culture medium (CM)). The cells were counted, distributed into minimum two aliqots and cultured as 10^6^ cells/mL CM with or without 1 *μ*g/mL LPS, in round-bottomed polypropylene vials (38 × 12.5 mm, Nunc, Roskilde, Denmark) in 5% CO_2_/humidified air at 37°C. LPS dose was chosen by dose effect of LPS in this system (0.1 ng/mL-1000 ng/mL). After 24 h, the incubation was terminated by centrifugation at 250 g and aliquots of supernatants were stored at −80°C until TNF*α* measurements. Shorter incubation time (3 hours) demonstrated much lower TNF production by PBMCs.

### 2.3. Bioassays of TNF*α*-Activity

Ten *μ*L aliquots of *serum samples* were diluted with 40 *μ*L of serum-free Minimum Essential Medium (MEM; Sigma) in 96-well tissue culture plates, and 50 *μ*L of suspension of WEHI-164 cells in serum-free MEM was added resulting in 2 × 10^4^ cells/well in 10% (patient's) serum containing fluid environment. For measuring TNF activity in *lymphomonocyte conditioned media*, 20 *μ*L aliquots of culture media taken from lymphomonocyte cultures (grown in 10% FCS supplemented MEM) were added to microcultures of WEHI-164 cells (2 × 10^4^ cells/well) growing in 80 *μ*L MEM medium supplemented with 10% fetal calf serum. Control cultures were grown in MEM supplemented with 10% FCS. For calibration, WEHI-164 cells (2 × 10^4^ cells/well) were incubated with various concentrations (0.005–10 ng/mL) of human TNF*α* under the same conditions. 

WEHI cells were incubated for 24 hours at 37°C with 5% CO_2_. At the end of the incubation, the viability of the cultures was determined by MTT-reduction method. Briefly, 50 *μ*L of the culture medium was aspirated and 10 *μ*L of 1.25 mg/mL stock solution of MTT in phosphate-buffered saline was added (final MTT concentration of 0.125 mg/mL). After 1.5 hour incubation, 150 *μ*L acidified (0.08 M HCl) isopropanol was added, and the produced formazan was dissolved by trituration. The optical adsorption was determined at 570 nm (measuring) and 630 nm (reference) wavelengths in a microplate reader (MWG-BIOTECH/BioRad). 

Each data point was determined as the mean ± SD of data obtained from 4 sister cultures. The viability was calculated as the percentage of the optical density of controls (100%). The viability values were converted to TNF*α* concentrations by the equation obtained by viability determinations at defined TNF*α* concentrations. 

TNF measurement of samples obtained from different subgroups of volunteers and sample taking were repeated in 3 months, 1 year and 9 years. 

Clinical laboratory values like lymphocyte counts, CRP, and *γ*GT were assessed by routine clinical procedures

### 2.4. Statistical Analysis

All data are presented as means ± standard deviations (SD). Statistical analysis was performed using Student *t*-test for inducibility of PBMCs. *P* < 0.05 was judged statistically significant.

## 3. Results

The demographic data and important laboratory values of volunteers included in the study are summarized in Tables 1 and 2. CRP and *γ*GT values were significantly higher in elderly volunteers than in younger ones. Despite the elevation, *γ*GT remained within the normal range, while CRP elevation was above the normal range. 

### 3.1. Elderly Volunteers

**Figure 1:**
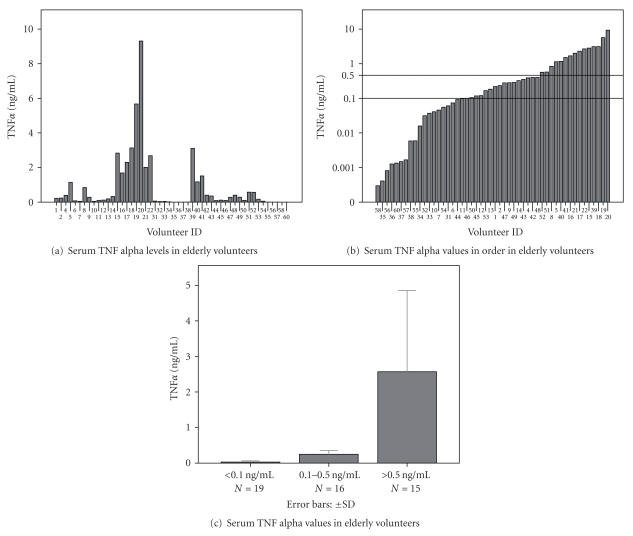
Serum TNF*α* individual values in elderly volunteers (a) and order of individual values (b) demonstrate three subgroups of the elderly population (c).

**Figure 2:**
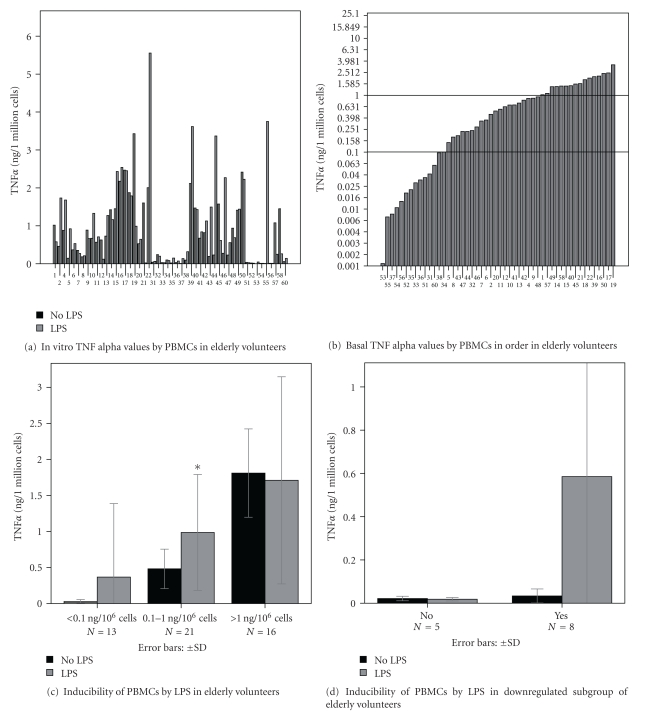
In vitro TNF*α* productions by peripheral blood mononuclear cells (PBMCs) of elderly volunteers demonstrate significant interindividual differences in this group (a) based on the order of TNF*α* production (b) three subgroups can be distinguished: downregulated, intermediate regulated, and upregulated (c) no response to endotoxin (LPS) was found in upregulated and in the majority of downregulated groups (c, d), *P < 0.05 stimulated versus nonstimulated.

From 60 volunteers, data of 50 persons were successfully analyzed. Noticeable individual differences were found in serum TNF*α* activity levels (Figures 1(a) and 1(b)). Ordering the individual data, three different subgroups could be postulated (c): groups of individuals with low, middle and high TNF-activities were distinguished. Similar marked individual differences were found in TNF*α* production by PBMCs (Figures 2(a) and 2(b)). In the majority of culture fluids of nonstimulated lympho/monocytes, TNF*α* activity between 0.1 ng and 1.0 ng/10^6^ cells/24 hours was found. In about 25% of cultures, however, significantly lower (<0.1 ng/10^6^ cells/24 hours) and in about 30% of preparations higher (>1.0 ng/10^6^ cells/24 hours) TNF-production was found (Figure 2(c)). Ordering the data resulted in a similar distribution of *in vitro* TNF-production as the distribution of serum TNF activity levels. In response to endotoxin (LPS) stimulation, the cytokine activity increased in the middle and in the majority of low TNF producing cultures, while high producers did not respond with enhanced TNF*α* production (Figure 2(c)). In 5 of 13 low producer cultures (producing less than 0.1 ng TNF*α*/10^6^ cells/24 hours), however, also failed to respond to endotoxin stimulation (d). Four of 5 of these volunteers died within 30 days after blood sampling due to pneumonia. 

### 3.2. Young Volunteers

**Figure 3:**
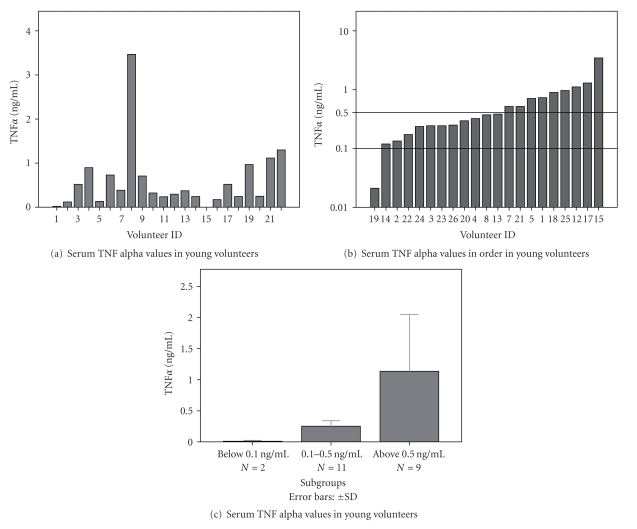
Serum TNF*α* individual values in young volunteers (a) order of individual values (b) demonstrate three subgroups of the young population (c).

**Figure 4:**
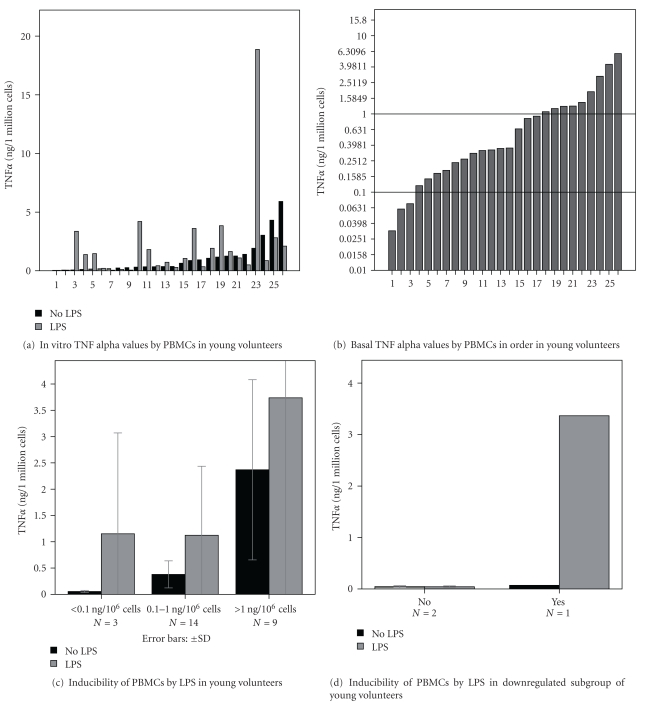
In vitro TNF*α* productions by peripheral blood mononuclear cells (PBMCs) of young volunteers demonstrate significant interindividual differences in this group (a) based on the order of TNF*α* production (b) three subgroups can be distinguished: downregulated, intermediate, and upregulated (c) no response to endotoxin (LPS) was found in the majority of downregulated group (d), *P < 0.05 stimulated versus nonstimulated.

Similarly to the elderly population, serum TNF*α* activities demonstrated large individual differences (Figures 3(a) and 3(b)) among young volunteers. Accordingly, three subgroups could be distinguished also in the young population (c). Individual differences in TNF*α* production by PBMCs were also found (Figures 4(a) and 4(b)) and low, middle, and high TNF-producer cultures (c) could be categorized. In the subgroup with lowest TNF*α* production (*n* = 3), lympho/monocytes of 2 volunteers did not respond to endotoxin stimulation (d). 

**Figure 5:**
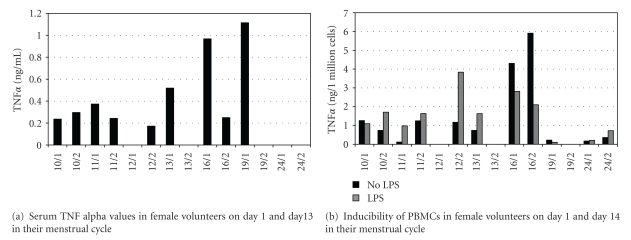
Female volunteers also demonstrate high interindividual differences both in circulating TNF*α* and inducibility of PBMCs during menstrual cycle. Interestingly, volunteer 16 demonstrates the highest TNF*α* values, and endotoxin tolerance can be observed as a part of the autoregulatory system protecting against extremely high TNF production.

As cytokine production changes with the menstrual cycle, blood samples of 7 young female volunteers were taken on day 1 and 14 of the cycle. The results varied with the cycle individually, both in serum and in *in vitro* experiments (Figures 5(a) and 5(b)) indicating similar interindividual differences in females as observed in males. These interindividual differences are complicated further by menstrual cycle.

Repeated measurements by the time gave the same amount of TNF and subgroups of individuals supported the idea that phenotype differences found is a constant quality of human population.

## 4. Discussion

The main findings of the study can be summarized as follow: (a) elderly and young human populations show marked individual differences in respect of circulating TNF*α* activity as well as in the production of TNF*α* by PBMCs; (b) the TNF*α* activity values outline down-, intermediate and upregulated subgroups in the investigated population, irrespective of age; (c) five individuals in the “downregulated” subgroup of elderly people did not respond to LPS stimulation, and four of them died of pneumonia within one month; (d) young females display TNF*α* activity fluctuations in connection with the menstrual cycle.

TNF*α* while serving host defense at appropriate concentrations, results in adverse systemic responses if present in excess. At low dose, it is essential; at higher dose, it is harmful [2, 10]. In the emerging era of personalized medicine, the significant interindividual differences in TNF production, those not found in other cytokines [11], should be taken into account. Marked interindividual differences in cytokine and cyclo-oxygenase production by human monocytes were published [3].

The observed individual differences in production of TNF*α*, but not in other cytokines [11], can be, in part, explained by gene polymorphisms. Several cytokine gene polymorphisms have been identified as factors in susceptibility to various diseases, including autoimmune, infectious, allergic, or cardiovascular diseases [10, 12, 13]. Polymorphisms in the regulatory regions of cytokine genes are associated with high and low cytokine production and may modulate the magnitude of alloimmune responses following transplantation [13]. The frequency of TNF*α* genotypes was also significantly different between multiple organ failure patients and controls. Intermediate TNF*α* producers were underrepresented (5.7% versus 23%), and high TNF*α* producers were overrepresented (35.2% versus 16%) in the patient group [14]. 

A genetic predisposition to high interleukin-10 production or intermediate TNF*α* production may be protective of admission to the intensive care unit although once admitted, any protection provided by these genotypes seems to be lost. The combination of proinflammatory and anti-inflammatory cytokine genotypes supports the idea that a balanced cytokine response is favorable and was associated with prolonged patient survival time [14]. 

Multiple genetic screening might forecast the potential cytokine responses, TNF*α* genotyping alone, however, will not predict it and will not correlate with mortality [14].

TNF*α* level and activity is strongly regulated by other cytokines. Cyclo-oxygenase metabolites such as PGE_2_ have been shown to inhibit TNF*α* production at high concentrations but seem to augment it at low concentrations. There is increasing evidence to suggest that the production of eicosanoids, PAF, and cytokines may be interrelated: IL-1 and TNF*α* induce PG synthesis in various cells, and PGs, in turn, modulate cytokine production [4]. In contrast, leukotriens can augment IL-1, IL-6, and TNF production and IL-1, TNF and IFN*γ* can also induce the synthesis of PAF in several cell types, including endothelial cells, neutrophils, and macrophages, while PAF can, in turn, augment IL-1, IL-6, and TNF production by rat and human cells. Such positive feedback loop with potential to amplify immune or inflammatory responses may be counterbalanced by the negative feedback action of IL-6 on both IL-1 and TNF [4]. This negative feedback may account for the limited production of IL-1 by LTB_4_-stimulated monocytes, which readily produce large amounts of IL-6. It may also explain the augmented production of IL-1 and TNF observed after treatment of monocytes with a dual CO/5-LO inhibitor [4]. 

The multiple regulations including the roles of soluble TNFRs beside cytokine crosstalks [8] inspired us to measure TNF*α* activity instead of the determination of the protein level itself. In the present study the necrotising effect of samples on WEHI 164 cells were determined, that is, a balanced effect of cell necrotizing and protective materials was investigated. Consequently, a more realistic biological activity was measured. 

When comparing young and elderly population endotoxin tolerance, for example, the fact that repeated LPS stimulation is less effective than the primary challenge in respect of TNF*α* production should not be neglected. The higher CRP values in elderly volunteers may indicate several inflammatory processes, including previous LPS challenges, during a long life story. While endotoxin tolerance is a well known situation, controversial data are available concerning its beneficial or harmful consequences [15]. At high TNF*α* production, the endotoxin tolerance may be beneficial because it can prevent further TNF*α* production. Our results, however, indicate that it can be detrimental, in cases when low TNF*α* activity is combined with low inducibility. 

The explanation of low inducibility observed in young volunteers is less clear. Even in the lack of clear explanation, however, the fact that a nonignorable part of people displays low TNF*α* activity combined with low TNF*α* inducibility urges clinical considerations. It means that there are individuals (about 10% of the investigated population) who are more endangered by infections, irrespective of age. Moreover, endotoxin tolerance may enlarge this danger with aging. 

## 5. Conclusion

Taken together, our results, in accord with earlier observations, strongly suggest the neccessity of rapid tests for determination and monitoring TNF levels. The controversial results of several anti-TNF treatments in human sepsis might be also explained by knowing the personal level of this pleiotropic cytokine at the start of treatment [16]. 

## Figures and Tables

**Table 1:** Demographic data of elderly and young volunteers.

	Elderly volunteers						Young volunteers					
			Male		Female				Male		Female	
	*N* = 50		*N* = 18		*N* = 32		*N* = 26		*N* = 19		*N* = 7	
	Mean	±SD	Mean	±SD	Mean	±SD	Mean	±SD	Mean	±SD	Mean	±SD
Age	75,4	13,1	71,5	14,9	77,5	11,8	28.1	6.0	37,5	6.0	25.4	3.4

**Table 2:** Laboratory variables of elderly and young volunteers.

	Elderly volunteers		Young volunteers		Significance
	Mean	±SD	Mean	±SD	
Ly	2,4	5,8	2,4	0,6	ns
G/L					

CRP	18	29	3,8	2,8	*P* < 0.002
mg/L					

*γ*GT	34	31	16,3	9	*P* < 0.004
U/L					
